# Sex differences in pre-incarceration mental illness, substance use, injury and sexually transmitted infections and health service utilization: a longitudinal linkage study of people serving federal sentences in Ontario

**DOI:** 10.1186/s40352-023-00218-9

**Published:** 2023-04-01

**Authors:** Tenzin Butsang, Arthur McLuhan, Leslie A. Keown, Kinwah Fung, Flora I. Matheson

**Affiliations:** 1 Dalla Lana School of Public Health, University of Toronto, Toronto, ON Canada; 2 MAP Centre for Urban Health Solutions, St. Michael’s Hospital, Toronto, ON Canada; 3 Research Branch, Correctional Service of Canada, Ottawa, ON Canada; 4 ICES, Toronto, ON Canada; 5 Centre for Criminology and Socio-Legal Studies, University of Toronto, Toronto, ON Canada

**Keywords:** Mental illness/substance use, Injury, Sexually transmitted infections, Health service utilization, Sex differences, Pre-incarceration

## Abstract

**Background:**

People who experience incarceration have poorer health than the general population. Yet, we know little about the health and health service utilization of people during the critical period prior to their incarceration, relative to during incarceration and post-release. In this study, we conducted a longitudinal cohort study of 39,498 adults in Ontario, Canada between January 1, 2002, and December 31, 2011 using linked administrative health and correctional data to describe mental illness, substance use, injury, sexually transmitted infections and health service utilization of men and women in federal prisons in the 3 years prior to their incarceration, compared to a matched group.

**Results:**

We found that, in the 3-year period prior to their incarceration, men (*n* = 6,134) and women (*n* = 449) experiencing their first federal sentence had poorer health across all indicators examined (e.g., psychosis, drug/alcohol use, and self-harm) and higher outpatient psychiatric and emergency department visits, compared with the matched group. Women in the pre-incarceration group exhibited a higher prevalence of self-harm and substance use, relative to women in the matched comparison group and higher relative prevalence to that of men in the pre-incarceration group, compared to their matched counterparts.

**Conclusions:**

Disparities in health and health service utilization are gendered and exist prior to incarceration. The gendered nature of these findings, specifically the significantly higher prevalence of poor health among women across several indicators, necessitates a focus on the social and systemic factors that contribute to these disparities. Gender-responsive and trauma-informed primary, secondary, and tertiary prevention strategies, alongside transformative approaches to justice should be considered in addressing the health needs of men and women who experience incarceration.

**Supplementary Information:**

The online version contains supplementary material available at 10.1186/s40352-023-00218-9.

## Background

People who experience incarceration have poorer health than the general population across an array of indicators, including chronic physical health conditions, infectious illnesses, traumatic brain injury, psychiatric and substance use disorders, self-harm, suicide, and mortality (Binswanger et al., 2009; Fazel & Baillargeon, 2011; Kouyoumdjian et al., 2016; Wilper et al., 2009). Research on pre-incarceration health has the potential to inform health services and improve health outcomes, during custody (e.g., intake screening procedures, diagnostic capacity, primary care resources) and post-release (e.g., discharge planning, reentry programs), but research on the pre-incarceration period is scarce (Leigey & Reed, 2010). The emerging research in the area has tended to rely on limited research data and designs such as small-N samples (Ramaswamy et al., 2015; Wilper et al., 2009), cross-sectional designs (Kouyoumdjian et al., 2015; Wilper et al., 2009), and self-reported health data (Conklin et al., 2000). Such designs have limited statistical power and do not allow for exploration of a temporal link between outcomes and risk factors (Hackshaw, 2008; Setia, 2016). In correctional research small samples are particularly problematic when examining gender/sex differences as women reflect only a minority of people who are incarcerated (Kouyoumdjian et al., 2016). Self-report measures are vulnerable to reporting errors, such as limited memory, selective disclosure, and incarceration-related changes to health awareness, such as intake assessments that reveal health conditions that were undiagnosed during the pre-incarceration period (Massoglia & Remster, 2019). A population-based cohort study design with relevant comparator groups allows us to better understand the needs of people entering prison and identify potential opportunities to improve carceral and community health services.

Relatedly, the incarcerated population comprises several subpopulations, each with a somewhat distinct set of health challenges and health care needs. For example, sex- and gender-differences are evident in pre-incarceration health status, behavior, and service utilization (Conklin et al., 2000). Still, most prisoner health research has focused on men (Kouyoumdjian et al., 2015), and as the number of women in prison continues to increase (Government of Australia, 2020; Kajstura, 2019; Sapers, 2015), research should attend to sex-related patterns of health and health service utilization among people with experiences of incarceration.

Several studies suggest that trauma is especially salient among women prior to incarceration (Bodkin et al., 2019; Leigey & Reed, 2010; Moore et al., 2021; Wolff & Shi, 2012). Leigey and Reed (2010) showed that women serving life sentences experienced significantly more traumatic events (e.g., abuse) prior to incarceration than life-sentenced males or females in the general population. Another study examining correlates of pre-incarceration health care use among men and women in jails found that more women visited primary care, emergency departments and were hospitalized in comparison to men; a higher percentage of women relative to men reported drug dependence and mental health diagnosis (Ramaswamy et al., 2015).

This study begins to address some of the critical gaps that remain in our knowledge of sex- and gender-related patterns of pre-incarceration health and health service utilization. Drawing on a record-linkage generated database that joined Canadian federal correctional data and administrative health data – “one of only a few population-based, longitudinal datasets to capture a wide range of health and correctional indicators from incarcerated individuals in the world” (McIsaac et al., 2016) – we compare the pre-incarceration mental illness, substance use, injury, sexually transmitted infections and health service utilization of men (*n* = 6,134) and women (*n* = 449) experiencing their first federal sentence during the study period in Ontario, Canada, relative to a matched comparison group. Among the pre-incarceration group we compare differences in the noted health indicators and health care utilization.

## Methods

### Setting

Correctional services in Canada fall under the jurisdiction of provincial, territorial, and federal governments. Individuals with sentences of less than two years are detained in provincial or territorial correctional facilities, while individuals sentenced to two or more years are held within federal institutions. Those incarcerated in federal penitentiaries are one of the few groups excluded by the *Canada Health Act* (Canada Health Act, 1985). Instead, health care services in federal prisons are legislated by the *Corrections and Conditional Release Act* (CCRA).

### Study design

We conducted a retrospective cohort study of adults in Ontario between January 1, 2002, and December 31, 2011, using secondary linked administrative data ﻿(McIsaac et al., 2016).

### Participants

For the pre-incarceration group, we included all men and women, age 18 and over, who experienced their first federal incarceration between January 1, 2005 and December 31, 2011 (*n* = 6,583; women = 449; men = 6,134). Participants included men and women admitted to any federal correctional facility in the province of Ontario, Canada, who were eligible for health care in Ontario for at least 2 years of a 3-year period prior to index date of first federal incarceration, as listed in the Registered Persons Database (RPDB), a population-based registry for health care maintained by the Ontario Ministry of Health and Long-Term Care. While this article uses gendered terms such as “men” and “women”, the administrative data used in this study characterize participants as “male” and “female” based on attributes that comprise biological sex. We excluded those who were either federally incarcerated but admitted to a facility in another province or were not federally incarcerated. We employed the former exclusion because we only had health information for persons eligible for health care in the province of Ontario. We created a 5:1 matched comparison group based on year of birth, sex and quintiles of the Ontario Marginalization Index (see descriptions in variables section) (van Ingen & Matheson, 2022). Controls (*n* = 530; 5:1 match) were assigned to each case with the same year of birth, sex, and material deprivation quintile, selecting randomly without replacement from the general population. Controls were retained if they had at least 730 days of OHIP (health insurance) eligibility in the 3 years prior to their assigned index date – the sentence commencement date of first federal sentence.

### Data sources and linkage

We linked administrative health care data with correctional records with a 98% linkage rate (McIsaac et al., 2016). Health databases included RPDB (for valid health cards), the Ontario Health Insurance Plan (OHIP; for physician visits), the Canadian Institute for Health Information (CIHI) Discharge Abstract Database (DAD; for hospital admissions), the CIHI National Ambulatory Care Reporting System (NACRS; for emergency department visits) and the Ontario Mental Health Reporting System (OMHRS; for inpatient mental health visits). The DAD provides patient data for acute, rehab, chronic, and day surgery institutions. OHIP provides information on patient ambulatory visits to physicians. The RPDB provides basic demographic information (age, sex) for anyone who has ever had an Ontario health card number. OMHRS provides information on stays on inpatient mental health beds. NACRS collects data information on patient visits to emergency departments. Diagnoses are based on the World Health Organization’s International Classification of Diseases, 9^th^ and 10^th^ Revisions (ICD 9 and 10) (World Health Organization, 2019).

The Correctional Service of Canada’s Offender Management System (OMS) provided computerized correctional records. This database includes information such as risk assessment, sentencing information, criminal history, and sociodemographic data on all adults in federal correctional facilities from their admission to sentence completion. These health and correctional datasets were linked using unique encoded identifiers and analyzed at ICES, an independent, non-profit research institute whose legal status under Ontario’s health information privacy law allows it to collect and analyze health care and demographic data, without consent, for health system evaluation and improvement. This project has been approved by the Research Ethics Board at [Name of institution where REB approval sought].

### Variable definitions

Age and sex were extracted from the RPDB (Table 1). Material deprivation – a measure of area-level marginalization in the Ontario Marginalization Index that can be used as a proxy for individual-level socioeconomic status – was used for matching. Material deprivation is one of 4 dimensions of the Ontario Marginalization Index which reflects marginalization in small geographic census areas (e.g., Census tract, Dissemination Area). Material deprivation is the dimension that is “conceptually connected to poverty and socio-economic position and associated with indicators of low income, educational attainment, quality of housing, and family housing characteristics” (van Ingen & Matheson, 2022, P.262). Marginalization is often associated with poor health and criminal justice involvement (Linton et al., 2017; Sariaslan et al., 2013). We captured residential postal code from the RPDB records (at time of incarceration) to link individuals to their neighborhood of residence and assign area-level material deprivation and rural/urban status based on Census data.

**Table 1:** Sociodemographic characteristics by group and sex, Ontario, Canada 2005–2011 (*N* = 39,498)

	**Overall**		**Women**		**Men**	
	Pre-incarceration Group *N* = 6,583	Matched Comparison Group *N* = 32,915	Pre-incarceration Group *N* = 449	Matched Comparison Group *N* = 2,245	Pre-incarceration Group *N* = 6,134	Matched Comparison Group *N* = 30,670
**Age**						
Mean ± SD	34.32 ± 11.52	34.33 ± 11.52	34.92 ± 10.23	34.92 ± 10.21	34.28 ± 11.61	34.29 ± 11.61
**Material Deprivation Quintile**						
1 (least deprived)	771 (11.7%)	3,855 (11.7%)	49 (10.9%)	245 (10.9%)	722 (11.8%)	3,610 (11.8%)
2	853 (13.0%)	4,265 (13.0%)	60 (13.4%)	300 (13.4%)	793 (12.9%)	3,965 (12.9%)
3	1,050 (16.0%)	5,250 (16.0%)	75 (16.7%)	375 (16.7%)	975 (15.9%)	4,875 (15.9%)
4	1,253 (19.0%)	6,265 (19.0%)	88 (19.6%)	440 (19.6%)	1,165 (19.0%)	5,825 (19.0%)
5 (most deprived)	2,386 (36.2%)	11,930 (36.2%)	155 (34.5%)	775 (34.5%)	2,231 (36.4%)	11,155 (36.4%)
Missing	270 (4.1%)	1,350 (4.1%)	22 (4.9%)	110 (4.9%)	248 (4.0%)	1,240 (4.0%)
**Residence**						
Urban	5,792 (88.0%)	28,934 (87.9%)	392 (87.3%)	1,977 (88.1%)	5,400 (88.0%)	26,957 (87.9%)
Rural	684 (10.4%)	3,930 (11.9%)	46 (10.2%)	264 (11.8%)	638 (10.4%)	3,666 (12.0%)
Unknown	107 (1.6%)	51 (0.2%)	11 (2.4%)	< = 5 (0.2%)	96 (1.6%)	47 (0.2%)

Three-year histories of health service utilization prior to incarceration, except for traumatic brain injury which is 5-year history

General population matched on age, sex, and Ontario Marginalization Index Material Deprivation Quintile

^*^IQR Interquartile range

Health was examined through TBI, self-harm, assault, accidents, sexually transmitted infections (STIs), alcohol/drug use, psychosis, and depression in the 3 years prior to index date of first incarceration (except TBI which was based on a 5-year look-back window) and defined as diagnoses based on the ICD 9 and 10 (see Supplemental Table 1) and from OHIP billing codes (for STIs). Health service utilization, in the 3-years prior to the index event of incarceration, was defined as emergency department, outpatient psychiatric and specialist, and general practitioner visits.

### Statistical analysis

We calculated sample characteristics by sex and exposure to incarceration as frequencies and proportions for categorical indicators and means and standard deviations for continuous indicators. We compare men in the pre-incarceration group to the matched comparison group; women in the pre-incarceration and matched comparison group; and men and women within their respective pre-incarceration groups. We present p-values and weighted standardized differences. All analyses were conducted in SAS v 9.4 (SAS Institute Incorporated, Cary, North Carolina, USA).

## Results

Table 1 presents the sociodemographic characteristics for the pre-incarceration (*N* = 6,583) and matched comparison (*N* = 32,915) groups by age, sex, and neighborhood material deprivation. The pre-incarceration group comprised 449 women and 6,134 men. The mean age at sentence commencement was 34.3 years (SD = 11.5) overall, 34.9 (SD = 10.2) for women and 34.3 (SD = 11.6) for men. Most people in the pre-incarceration group lived in urban areas (*n* = 5,792 [88%]) and in neighborhoods with high material deprivation, with 3,639 (55.2%) located in the two most deprived quintiles (Q4, Q5), as per the Ontario Marginalization Index.

Table 2 presents health indicators for the pre-incarceration and matched comparison groups for the overall sample and by sex. Relative to the matched comparators, men and women in the pre-incarceration group were significantly more likely than the matched comparison group to experience a higher prevalence of all health indicators. Pre-incarceration group women and men had much higher rates of injury than women and men in the matched comparison groups, respectively. In the five years prior to incarceration, pre-incarceration women were about five times more likely than matched comparison women to have a TBI, and men in the pre-incarceration group were about four times more likely to have a TBI than men in the matched comparison group. In the three years prior to incarceration, the prevalence of self-harm among pre-incarceration group women was more than 14 times the rate among matched comparison women, and self-harm among men in the pre-incarceration group was more than 11 times the rate among men in the matched comparison group. The three-year history of assault reveals similar disparity between the pre-incarceration and matched comparison groups, with assault being about 17 times more likely among pre-incarceration women and nearly 8 times more likely among pre-incarceration men. Accidents were also high, being more than double among women in the pre-incarceration group compared with women in the matched comparison group, and more than 1.5 times among men in the pre-incarceration group compared with men in the matched comparison group. In the pre-incarceration groups, sexually transmitted illness was more than four times higher among women and nearly 3.5 times higher among men compared with their respective matched comparison groups.

**Table 2:** Pre-incarceration health by group and sex, Ontario, Canada (*N* = 39,498)

	**Overall**			**Women**			**Men**			**Pre-incarceration** **Women vs. Men**
	**Pre-incarceration Group *N* = 6,583**	**Matched Comparison Group *N* = 32,915**	**Weighted Standardized Difference**	**Pre-incarceration Group *N* = 449**	**Matched Comparison Group *N* = 2,245**	**Weighted Standardized Difference**	**Pre-incarceration Group *N* = 6,134**	**Matched Comparison** **Group** **N = 30,670**	**Weighted** **Standardized** **Difference**	**Weighted** **Standardized** **Difference**
**Injuries**										
TBI	744 (11.3%)	957 (2.9%)	0.331^***^	44 (9.8%)	46 (2.0%)	0.333^***^	700 (11.4%)	911 (3.0%)	0.331^***^	0.05
Self-harm	324 (4.9%)	136 (0.4%)	0.283^***^	45 (10.0%)	16 (0.7%)	0.422^***^	279 (4.5%)	120 (0.4%)	0.270^***^	0.21^***^
Assault	1,008 (15.3%)	625 (1.9%)	0.493^***^	54 (12.0%)	16 (0.7%)	0.476^***^	954 (15.6%)	609 (2.0%)	0.494^***^	0.10^*^
Accident	2,741 (41.6%)	8,031 (24.4%)	0.373	178 (39.6%)	379 (16.9%)	0.522^***^	2,563 (41.8%)	7,652 (24.9%)	0.363^***^	0.04
**Sexually Transmitted Infection**	677 (10.3%)	956 (2.9%)	0.301^***^	55 (12.2%	64 (2.9%)	0.362^***^	622 (10.1%)	892 (2.9%)	0.296^***^	0.07
**Mental Illness & Substance Use**										
Alcohol use	1,126 (17.1%)	874 (2.7%)	0.499^***^	84 (18.7%)	27 (1.2%)	0.611^***^	1,042 (17.0%)	847 (2.8%)	0.491^***^	0.04
Drug use	2,244 (34.1%)	1,201 (3.6%)	0.844^***^	211 (47.0%)	70 (3.1%)	1.174^***^	2,033 (33.1%)	1,131 (3.7%)	0.822^***^	0.29^***^
Psychosis	711 (10.8%)	491 (1.5%)	0.395^***^	48 (10.7%)	22 (1.0%)	0.423^***^	663 (10.8%)	469 (1.5%)	0.393^***^	0.00
Depression	2,237 (34.0%)	2,318 (7.0%)	0.708^***^	177 (39.4%)	234 (10.4%)	0.712^***^	2,060 (33.6%)	2,084 (6.8%)	0.708^***^	0.12^**^

Three-year histories of health service utilization prior to incarceration, except for traumatic brain injury which is 5-year history

General population matched on age, sex, and Ontario Marginalization Index Material Deprivation Quintile

^***^p ≤ 0.001; ^*^p ≤ 0.01; ^*^p ≤ 0.05

Table 2 also presents the history of mental illness and substance use in three years prior to incarceration and shows statistically significant differences between pre-incarceration and matched comparison groups. Both pre-incarceration group women and men had much higher rates of mental illness and substance use than their respective matched samples. Alcohol use was more than 15 times higher among women and six times higher among men in the pre-incarceration groups relative to their respective matched comparison groups. Similarly, drug use was about 15 times more prevalent among women and nearly 9 times more prevalent among men in the pre-incarceration groups, relative to the respective matched comparison groups. Psychosis was nearly 11 times higher among women and more than 7 times higher among men in the pre-incarceration groups, relative to the matched comparison groups. Depression was more than 3.5 times higher among pre-incarceration women and approximately five times higher among men in the pre-incarceration groups compared with respective matched comparison groups.

When comparing within the pre-incarceration group (see standardized differences and *p*-value in the last column of Table 2), women were 2.2 times more likely to experience self-harm, almost 1.5 times more likely to experience drug use, and 1.2 times more likely to experience depression relative to men. Men, relative to women in the pre-incarceration group were 1.3 times more likely to experience assault.

Table 3 presents health care utilization in the three years prior to incarceration. With the exception of outpatient specialist visits, women and men in the pre-incarceration groups had significantly higher rates of health service visits relative to respective matched comparison groups. For emergency department visits, pre-incarceration women averaged 3.6 visits compared with 1.2 visits for women in the matched comparison group, and pre-incarceration men averaged 2.4 visits compared with 0.9 visits for those in the matched comparison group. For outpatient psychiatrist visits, pre-incarceration women averaged 2.7 visits compared with 0.4 visits among women in the matched comparison group, and men in the pre-incarceration group averaged 2.6 visits compared with 0.4 visits for men in the matched comparison group. Outpatient specialist visits were comparable across groups, with pre-incarceration group women averaging 3.9 visits compared with 4.4 visits for matched comparison group women, and men in the pre-incarceration group averaging 1.9 visits compared with 1.8 visits for men in the matched comparison group. For general practitioner visits, women in the pre-incarceration group averaged 26.3 visits compared with 12.0 for women in the matched comparison group, while pre-incarceration group men averaged 17.9 visits compared with 6.7 for men in the matched comparison group.

**Table 3:** Pre-incarceration health service utilization by group and sex, Ontario, Canada (*N* = 39,498)

		**Overall**			**Women**			**Men**			**Pre-incarceration Women vs. Men**
		**Pre-incarceration Group *N* = 6,583**	**Matched Comparison Group N = 32,915**	**Weighted Standardized Difference**	**Pre-incarceration Group N = 449**	**Matched Comparison Group N = 2,245**	**Weighted Standardized Difference**	**Pre-incarceration Group N = 6,134**	**Matched Comparison Group N = 30,670**	**Weighted Standardized Difference**	**Weighted Standardized Difference**
**Health Service Utilization**											
Emergency Dept. visits (unplanned)	Mean ± SD	2.46 ± 5.30	0.95 ± 2.15	0.37^***^	3.54 ± 5.84	1.19 ± 4.01	0.47^***^	2.38 ± 5.25	0.93 ± 1.95	0.37^***^	0.21^***^
	Yes	4,199 (63.8%)	13,459 (40.9%)	0.47^***^	331 (73.7%)	989 (44.1%)	0.63^***^	3,868 (63.1%)	12,470 (40.7%)	0.46^***^	0.23^***^
Outpatient Psychiatrist visits	Mean ± SD	2.62 ± 8.47	0.39 ± 4.07	0.34^***^	2.69 ± 8.19	0.38 ± 3.21	0.37^***^	2.61 ± 8.49	0.39 ± 4.12	0.33^***^	0.01
	Yes	2,099 (31.9%)	1,352 (4.1%)	0.78^***^	164 (36.5%)	111 (4.9%)	0.85^***^	1,935 (31.5%)	1,241 (4.0%)	0.77^***^	0.11^**^
Outpatient Specialist visits	Mean ± SD	2.00 ± 5.61	1.97 ± 4.69	0.03	3.88 ± 7.26	4.38 ± 6.74	0.03	1.86 ± 5.44	1.79 ± 4.45	0.04	0.31^***^
	Yes	2,877 (43.7%)	13,891 (42.2%)	0.01^*^	273 (60.8%)	1,402 (62.4%)	0.07	2,604 (42.5%)	12,489 (40.7%)	0.01	0.37^***^
GP/FP outpatient visits	Mean ± SD	18.43 ± 24.15	7.02 ± 11.11	0.61^***^	26.33 ± 27.74	11.98 ± 13.73	0.66^***^	17.85 ± 23.76	6.66 ± 10.81	0.61^***^	0.33^***^
	Yes	6,282 (95.4%)	26,463 (80.4%)	0.47^***^	–-	2,025 (90.2%)	0.40^***^	5,837 (95.2%)	24,438 (79.7%)	0.48^***^	0.24^***^

Three-year histories of health service utilization prior to incarceration, except for traumatic brain injury which is 5-year history

General population matched on age, sex, and Ontario Marginalization Index Material Deprivation Quintile

^***^p ≤ 0.001; ^*^p ≤ 0.01; ^*^p ≤ 0.05

Within the pre-incarceration group, women averaged 3.54 and men 2.38 emergency department visits. Mean pre-incarceration specialist visits were 3.88 among women and 1.86 among men. Mean general practitioner visits was 26.33 for pre-incarceration women and 17.85 for pre-incarceration men. There was no significant difference in psychiatric visits between pre-incarceration men and women.

## Discussion

This study examined sex differences in pre-incarceration health status and health service utilization between people serving sentences of 2 or more years in Ontario, Canada and a comparison sample matched by sex, date of birth and material deprivation (a proxy for individual-level socioeconomic status). The findings from this paper suggest that injuries, including assault, self-harm, accidents, traumatic brain injury; sexually transmitted infection; mental illness; and substance use reign high for both pre-incarceration women and men in relation to the matched comparison group. Consistent with previous pre-incarceration health research (Fazel & Baillargeon, 2011; Kouyoumdjian et al., 2016, 2018; Leigey & Reed, 2010), our findings suggest that poor health is coupled with high use of emergency departments and outpatient psychiatric and specialist care for people prior to incarceration versus the matched comparison group. Pre-incarceration women specifically, had significantly higher utilization of all health services examined, except for outpatient psychiatric visits in comparison to their male counterparts. In comparisons between men and women in the pre-incarceration group, women had higher prevalence of self-harm, depression, and drug use, while pre-incarceration men – relative to women pre-incarceration women – had a significantly higher prevalence of assault. Our findings reinforce previous research on the high prevalence of exposure to assaultive violence (trauma) among incarcerated men (Wolff et al., 2014) and extends knowledge in this area by confirming the existence of these disparities prior to incarceration. The gendered nature of these findings, specifically the significantly higher prevalence of poor health among women across several indicators, necessitates a focus on the social and systemic factors that contribute to these disparities.

Relational theory, with additional insights from pathways, addiction, and trauma theories, can help us understand women’s trajectories to prison (Bylington, 1997; Covington, 2007; Steffensmeier & Allan, 1996). Diverging from early theories of development which emphasized individuation and autonomy as markers of self-actualization, Miller (1976) and Gilligan (1993) and the Stone Center Model (Jordan et al., 1991) argued that women’s contextual and relational experiences are key factors to their wellbeing. Building upon this early work, relational theory has three central tenets: culture deeply affects women’s lives; relationships – notably connections to others – are key in shaping women’s development; and connections (and emotions) have the potential to move women to healthy growth. It is in the context of this understanding of women’s development that we can begin to appreciate the impact of difficult relationships on women’s lives and how this may shape pathways to imprisonment.

If women self-actualize in connection with others and experience trauma in various forms including poverty and violence (as children and/or adults) – the latter in relation to their closest connections – then they are at greater risk of developing mental health concerns and to use substances as an escape from negative emotions. According to the pathways model (Steffensmeier & Allan, 1996) women enter into crime in response to experiences of violence and poverty. Trauma-informed approaches recognize that women’s relationships profoundly affect their life trajectories including mental health and substance use and that their crimes are motivated by survival and/or by abusive and neglectful relationships. Research suggests that women who experience incarceration are more likely than the general population to have histories of trauma, specifically physical and sexual abuse dating back to childhood, and that intimate partner violence and sex-trade related violence are often ongoing concerns in adult life (Baumann et al., 2019; Sorbello et al., 2002). These experiences shape women’s everyday orientations, perspectives, identities, emotions, and relationships (Covington & Bloom, 2007). The findings of the current study suggest that prior to experiencing incarceration, women have histories of accidents, injuries, substance use, mental health concerns and self-harm.

Attending to the health needs of men – specifically those made vulnerable by systemic factors such as poverty and criminalization – through the lens of relational theory and the pathways model can generate novel insights into the health disparities they experience. While men and women share several common pathways to prison, including a history of drug use or drug dealing and street involvement, these pathways are also gendered (Daggett, 2014). For example, Daggett (2014) found that women in contact with the criminal justice system were street-involved primarily due to fleeing abusive homes while men came to the streets from dropping out of school or quitting their job. Understanding men’s health in the context of their pathways to prison holds significant implications for gender-responsive care for both men and women. Considering these pathways alongside a relational theory of men’s health further illuminates the findings of this study. Men in the pre-incarceration group had significantly lower health care utilization than pre-incarceration women across all indicators except outpatient psychiatric visits. Courtenay (2000) suggests that the various social and institutional structures people encounter elicit different demonstrations of health behaviors and beliefs – for men, this often means a rejection of behaviors that are socially constructed as feminine in pursuit of dominant hegemonic masculine ideals. Further explorations of class, race, ethnicity, sexual orientation, and social context through a relational lens could yield critical contributions to our understanding of the health of both men and women who come in contact with the criminal justice system.

Despite similarly elevated rates of outpatient psychiatric visits among men and women in the pre-incarceration group, women in the pre-incarceration group had a significantly higher prevalence of depression, self-harm, and drug use compared to women in the matched comparison group. The significantly higher prevalence of self-harm among pre-incarceration women in our study aligns with recent findings on the increase of reported incidents of self-harm among women in prison (Syal, 2021). Given what is known about women’s development, neither the health nor the correctional system are well-equipped to meet the needs of these women without the implementation of gender-responsive and trauma-informed programs and services, particularly in relation to mental health and psychiatric services (Covington & Bloom, 2007). Covington and Bloom (2007) outlined five guiding principles for gender-responsive policy and programs designed to support women at any stage of criminal justice involvement. Such programs need to recognize that pathways to incarceration differ by gender; that safety, respect and dignity are essential components of policy and programs for women to support and enhance connections to their children, significant others and the community; and that services must be holistic, culturally relevant, and enhance women’s ability to be financially self-reliant. Boppre (2019) also argued that recognizing intersectional identities are critically important when addressing the needs of women facing incarceration given the overrepresentation of racialized women in the North American prison population.

### Strengths and limitations

A major strength of this study is its use of longitudinal population-level data for federally sentenced adults from the Correctional Service of Canada’s Offender Management System and the health administrative databases housed at ICES, rather than cross-sectional survey data. The study also benefitted from the use of clinical diagnoses for the health indicators of TBI, assault, injuries, self-harm, accidents, sexually transmitted infection, mental health, and substance use disorders. One potential limitation of the health data is that we captured only those diagnosed through a visit to the hospital or a physician. We are potentially underestimating these health conditions, for research suggests that there is reluctance on the part of this population to access health services, especially among men, for reasons including experiences of discrimination (Baumann et al., 2019; Frank et al., 2014; Pinkhasov et al., 2010). In a recent systematic review, Capon et al. (2020) maintained that stigma related to criminal justice involvement can act as a barrier to healthcare utilization; also noted by others (Baumann et al., 2019; Hu et al., 2020). Men may be particularly reluctant to seek health care, as it may be viewed as a sign of weakness (Courtenay, 2000; Galdas, 2009; Jeffries & Grogan, 2012). Research shows that men are less likely to access healthcare services than women, (Bertakis et al., 2000; Galdas, 2009). Given what is known about the health care utilization of people involved in the criminal justice system, the findings in this paper are likely conservative. While administrative health data has flaws, it is often considered superior to collection data through surveys which are prone to reporting and recall bias (Ties Boerma & Sommerfelt, 1993). The data does not attend to previous incarceration in the provincial correctional system and the study results may not be generalizable to jurisdictions with different health care systems and funding models.

The administrative data used in this study characterize participants as “male” and “female” based on attributes that comprise biological sex. While this article uses gendered terms such as “men” and “women”, the administrative data used in this study may not accurately represent an individual’s gender identity. Sex and gender are important determinants of health for people involved in the criminal justice system. Gender influences factors such as access to health care, use of the health care system and the behavioral attitudes of medical professionals toward those seeking care (Regitz‐Zagrosek, 2012) and biological attributes of sex such as hormone function have been positively associated with incarceration (Horn et al., 2014). To optimize health, well-being and successful reentry, the complex intersection of sex and gender should be factored into targeted supports for complex health and social needs. The data also did not allow us to examine differences in outcomes by race or ethnicity. In Canada, Indigenous and African, Caribbean and Black communities are overrepresented in both the federal and provincial correctional systems as a result of structural and systemic factors including racism, discrimination, and the legacy of colonization (Cardoso, 2020). This paper does not address these key determinants of health and incarceration in its analysis and should be a priority for future research in this area.

## Conclusions

While existing data highlights disparities in health and health service utilization among individuals during and post-incarceration (Binswanger et al., 2009; Kouyoumdjian et al., 2018; Wilper et al., 2009), our data illustrates that these disparities are gendered and exist prior to incarceration. Our findings indicate that women shoulder a disproportionate burden of health challenges prior to incarceration relative to their matched comparison counterparts. Existing health challenges prior to incarceration can be exacerbated in prison (De Viggiani, 2007) and can affect re-entry success (Cnaan et al., 2008). With increasing rates of incarceration among women worldwide (Walmsley, 2006), wellbeing among this target population should be a public health imperative. Gender-responsive public health solutions are urgently needed across individual, community, and system level interventions.

Evidence that both men and women have poorer mental health prior to incarceration compared to their counterparts in the non-incarcerated population suggests that at the individual level, facilitating access to gender-responsive and trauma-informed care should be a focus for health care and social service providers as part of the first steps toward addressing unmet health needs among this population, especially substance use and mental health concerns. At the community level, funding community-based services has the potential to support individuals and their families before they enter a cycle of poverty, poor health, and incarceration. With over half of individuals in the pre-incarceration group living in neighborhoods with high material deprivation, the community level implications of this study also include efforts to improve community infrastructures such as education, housing and employment.

Findings from this study also provide evidence for the critical need for systems level interventions to address disparities in health status and service utilization among men and women who experience incarceration. By elucidating the health of individuals entering prison with heavy health burdens, our findings highlight the importance of policies and institutional standards of care to ensure equitable access to health services for people while they are incarcerated, particularly in the areas of mental health and substance use treatment. Given the mass incarceration of Indigenous and African, Caribbean, and Black people across North America, due consideration of the unique needs of these communities is vital to understand and address the specific relationship between systemic racism and health and criminalization. Priorities for policy and program implementation for this population in both federal correctional institutions and provincial health care systems should be developed with the expertise of individuals with experiences of incarceration. Importantly, our findings highlight the need for non-punitive responses to the conditions of poverty, mental health, and substance use that place individuals in contact with the criminal justice system. Evidence-based alternatives to imprisonment, such as restorative and transformative justice initiatives (Pont et al., 2021), should be a central focus in breaking the cycle of poor health and incarceration.

## Supplementary Information

**Additional file 1:** **Supplementary table 1.**

## Data Availability

Health databases included Registered Persons Database (RPDB), a population-based registry for health care maintained by the Ontario Ministry of Health and Long-Term Care, the Ontario Health Insurance Plan (OHIP; for physician visits), the Canadian Institute for Health Information (CIHI) Discharge Abstract Database (DAD; for hospital admissions), the CIHI National Ambulatory Care Reporting System (NACRS; for emergency department visits) and the Ontario Mental Health Reporting System (OMHRS; for inpatient mental health visits). The Correctional Service of Canada’s Offender Management System (OMS) provided computerized correctional records. These health and correctional datasets were linked using unique encoded identifiers and analyzed at ICES, an independent, non-profit research institute whose legal status under Ontario’s health information privacy law allows it to collect and analyze health care and demographic data, without consent, for health system evaluation and improvement. The data that support the findings of this study are available from ICES (https://www.ices.on.ca/) but restrictions apply to the availability of these data, which were used under license for the current study, and so are not publicly available. Data are however available from the authors upon reasonable request and with permission of ICES.
