# Supplementary material for: Sex differences in pre-incarceration mental illness, substance use, injury and sexually transmitted infections and health service utilization: a longitudinal linkage study of people serving federal sentences in Ontario
Source: Health Justice. 2023 Apr 1;11:19. doi: 10.1186/s40352-023-00218-9 (PMC10067244; doi:10.1186/s40352-023-00218-9)
Supplement: Supplementary file 1 — Additional file 1: Supplementary table 1. [file 40352_2023_218_MOESM1_ESM.docx]

Supplementary Table 1: Health Related Definitions

| Variable | ICD-10 diagnosis codes* in hospital discharge, mental health records, and emergency department visit records | ICD-9 diagnosis codes in hospital discharge, mental health records, and emergency department visit records | ICD-9 diagnosis codes in physician claims |
| --- | --- | --- | --- |
| Assault | X85 – Y09 |  |  |
| Intentional Self-Injury | X60 – X84 |  |  |
| Traumatic Brain Injury | S020 S021 S023 S027 S028 S029 S06 S071 S098 S099 T902 T905 | 800 801 803 804 850 851 852 853 8540 8541 95901 | 803, 850, 854 |
| Accident | V01-X59 |  |  |
| Sexually Transmitted Infection | A50-A64 |  | 097, 098, 099 |
| Alcohol Abuse | F10, E52, G621, I426, K292, K700, K703, K709, T51, Z502, Z714, Z721 | 2552, 2911, 2912, 2913, 2915, 2916, 2917, 2918, 2919, 3030, 3039, 3050, 3575, 4255, 5353, 5710, 5713, 980, V113 | 291, 303 |
| Drug Abuse | F11, F12, F13, F14, F15, F16, F18, F19, Z715, Z722 | 292, 304, 3052, 3053, 3054, 3055, 3056, 3057, 3058, 3059, V6542 | 292, 304 |
| Psychoses | F20, F22, F23, F24, F25, F28, F29, F302, F312, F315 | 2938, 295, 29604, 29614, 29644, 29654, 297, 298 | 293, 295, 297, 298 |
| Depression | F204, F313, F314, F315, F316, F317, F318, F319, F32, F33, F341, F412, F432 | 2622, 2963, 2965, 3004, 3009, 311 | 296, 301, 311 |

*All ICD-10 diagnosis codes starting with the characters specified in the table were included in the definitions. All DSM diagnosis codes starting with the digits specified in the table were included in the definitions.
